# Evaluating the Food Profile in Qatar within the Energy–Water–Food Nexus Approach

**DOI:** 10.3390/foods12020230

**Published:** 2023-01-04

**Authors:** Odi Fawwaz Alrebei, Tareq Al-Ansari, Mohammad S. Al-Kuwari, Abdulkarem Amhamed

**Affiliations:** 1 Qatar Environment and Energy Research Institute (QEERI), Hamad Bin Khalifa University, Doha 34110, Qatar; 2 College of Science and Engineering, Hamad Bin Khalifa University, Qatar Foundation, Doha 34110, Qatar; 3 Environmental and Municipal Studies Institute, Ministry of Municipality and Environment, Doha 7634, Qatar

**Keywords:** energy–water–food nexus, self-sustainability, food security, water security, resource management

## Abstract

Finding a balance between the capacity for production and the rising demand for food is the first step toward achieving food security. To achieve sustainable development on a national scale, decision-makers must use an energy, water, and food nexus approach that considers the relationships and interactions among these three resources as well as the synergies and trade-offs that result from the way they are handled. Therefore, this paper evaluates the Energy–Water–Food Nexus Profile of Qatar at a superstructural level by applying the Business-As-Usual (BAU) storyline; thus, trends of past data have been used to provide future projections to 2050 using the statistical prediction tools such as the compound annual growth rates of food demand (${CAGR}_{FD}$), international supply (${CAGR}_{FI}$), and the average local food supply change factor ($\bar{c}$). Once the BAU storyline has been generated, the source-to-demand correlations have been defined for each food category. Such correlations include the annual and average ratios of the local food supply to the total demand (i.e., $\alpha_{i}$ and $\bar{\alpha }$) and the ratios of the local food supply to the international supply (i.e., $\beta_{i}$ and $\bar{\beta }$). In addition, as an effort to identify the required action to reach food self-sustainability, the additional local food supply to achieve ($x_{i,add})$ and its ratio to the local demand (γ) have been defined. The highest average ratio of the local food supply to the total demand ($\alpha_{i}$) was found for the meat category, which was estimated to be 48.3%. Finally, to evaluate the feasibility of attaining food self-sustainability in Qatar, the water consumption ($V_{w,i}$) and its corresponding required energy for each food category have been estimated.

## 1. Introduction

A deeper understanding of the connections and trade-offs is required for the energy–water–food nexus method. There are complicated and multi-directional links between water, energy, and food via various sub-nexuses. Academics and developers have shown the most interest in the water/energy nexus concept. Water use, both consumptive and non-consumptive, is strongly linked to energy production [1].

To produce, distribute, and treat water, significant energy is required for water pumping, irrigation, treatment, and desalination [2,3,4]. In addition, energy footprints vary for each water system. According to the estimates of the energy intensity of several water sources, desalinated water costs more than 4000 kilowatt hours per acre-foot. In comparison, local and recycled wastewater sources cost less than 500 kilowatt hours per acre-foot (326,000 L) of water [3]. Due to the underestimation of the variance in the energy footprints depending on the type of the water systems, large-scale water systems were designed and built during the hydraulic mission of the twentieth century but often failed to effectively account for varying energy needs [3].

The second part of the nexus emphasizes the connection between food and water. Over 70% of the freshwater on the planet is utilized for agriculture. Apart from the percolation and reflow into aquifers and rivers, a substantial fraction of this withdrawal is taken up by plants via evapotranspiration. However, while cooling water from a power plant may still be used for various purposes in the future, it can only be used once throughout a hydrological cycle. Consequently, agriculture accounts for far higher water consumption (i.e., 92 percent, compared to other applications [4,5]). For example, to visualize the food-to-water link, a ton of wheat needs 1300 cubic meters of water [6,7].

The third level of the nexus concept is focused on the sub-nexus of energy and food production. The energy system provides direct inputs to crops, livestock, fisheries, food processing, distribution, retail, preparation, and cooking [8,9,10]. Globally speaking, agriculture has grown significantly more dependent on fossil fuels during the 20th century. While fossil fuels have been widely utilized to drill wells, power tractors, and combine harvesters, the Haber–Bosch technique, invented in 1908–1910, enabled the industrial manufacture of nitrogen fertilizers [11,12,13,14,15,16,17,18,19,20].

Understanding customers, their food choices, and food waste is also necessary to comprehend the interaction between water, energy, and food. It takes more food, water, and energy to feed growing people. The increase of affluent consumers results from the world’s economic turmoil, particularly in Asia. Consequently, additional demands on the food, energy, and water systems are becoming more and more apparent [15,16,17,18,19,20,21,22,23,24,25].

Okonkwo et al. [26] investigated the potential of nanomaterials to improve the performance of the energy–water–food nexus as part of the endeavor to improve it. For the EWF nexus in the state of Qatar, Okonkwo et al. [26] presented an evaluation and reaction option. A comprehensive energy, water, and food (EWF) nexus strategy, incorporating the integration of sub-systems denoting EWF resources, was presented by Namany et al. [27]. In addition, Namany et al. [27] proposed a unique technique that applies EWF nexus thinking to decision-making in the agricultural industry while considering the uncertainty of energy costs produced from natural gas. In their study of the effects of local and regional climate changes, Hazrat et al. [28] identified the main driving factors that have altered the state of Qatar’s local groundwater resources and its implications for the energy, water, and food nexus. In addition, Lahlou et al. [29] developed a water planning framework for alfalfa fields utilizing treated wastewater fertigation in Qatar—an energy–water–food nexus approach. This was conducted in an attempt to advance the water profile in Qatar under the NEXUS approach. An innovative energy, water, and food nexus “Node” methodology was introduced in a study by Haji et al. [30] that included (a) decentralization using GIS-based approaches, (b) creation of composite geospatial risk indicators using the Analytical Hierarchy Process, and (c) evaluation of resource utilization. A flexible computational framework was suggested to describe the interconnections between the energy, water, and food (EWF) sectors in the study by Govindan, R., and Al-Ansari [31]. An integrated energy, water, and food life cycle assessment method was published by Al-Ansari et al. [32] to examine the environmental effects of increasing food production in Qatar. Research shows that food production is the most significant cause of global warming [32]. Al-Ansari et al. [33] offered an energy, water, and food nexus method based on this discovery to improve food production systems by CO_2_ fertilization. A study of carbon capture and use as a CO_2_ abatement possibility within the EWF nexus was also provided by Ghiat, Ikhlas, and Tareq Al-Ansari [34]. A targeted study of decision-making tools for effective resource management and governance was also carried out by Namany et al. [35].

Food production in Qatar significantly rose as a result, and it quickly became apparent that agricultural output was both an economically feasible and far more sustainable alternative. As a result, agriculture in Qatar is now seen as a developing sector that contributed to 0.2% of the country’s GDP in 2019 [12]. To meet the nation’s food demand, Qatar strongly supports exploring alternate supply-side approaches and sources.

Therefore, in response, this paper evaluates the Energy–Water–Food Nexus Profile of Qatar at a superstructural level by applying the Business-As-Usual (BAU) storyline; thus, trends of past data have been used to provide future projections to 2050 using the statistical prediction tools such as the compound annual growth rates of food demand ($CAGR_{FD}$), international supply ($CAGR_{FI}$), and the average local food supply change factor ($\bar{c}$).

The novelty of this paper appears in evaluating and providing future projections of the food profile in Qatar within the energy–water–food nexus approach, thus, providing decision-making data that can be used as a baseline to enhance the food sector to achieve a higher level of food security. Therefore, key mathematical indicators have been proposed and evaluated within this frame to provide correlations between the demand and supply categories. Such correlations include the annual and average ratios of the local food supply to the total demand (i.e., $\alpha_{i}$ and $\bar{\alpha }$) and the ratios of the local food supply to the international supply (i.e., $\beta_{i}$ and $\bar{\beta }$). In addition, to identify the required action to reach food self-sustainability, the additional local food supply to achieve ($x_{i,add})$ and its ratio to the local demand (γ) have been defined.

## 2. Methodology

### 2.1. Food Demand Profiling

Utilizing the raw data of food demand (Table 1) provided by the Planning and Statistics Authority of Qatar from 2015 to 2020 [36], the compound annual growth rate of food demand ($CAGR_{FD}$) has been estimated using Equation 1 [37] for all food categories. EV, BV, and n are the end value, beginning value, and the number of years, respectively.

(1) $$CAGR_{FD}={\left(EV/BV\right)}^{1/n}-1$$

**Figure 1:**
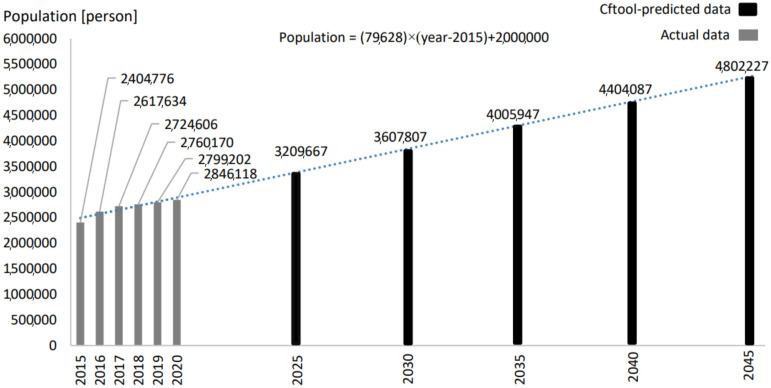
The actual population data (2015–2020) and the Cftool-projected population.

As shown in Table 1, the food demands for all categories have increased from 2015 to 2020. The compound annual growth rate of food demand is the highest for the fruits category, followed by the egg, meat, dairy products, vegetables, fish, and cereals categories, respectively. The overall increasing trend of the food demand is directly attributed to the increase in the population in Qatar, which increased from 2,437,790 persons in 2015 to 2,846,118 in 2020 (i.e., increased by 14.3%) [38] (Figure 1). Figure 1 shows the actual population from 2015 to 2020. In addition, utilizing the raw data of Qatar population (Table 1) provided by the Planning and Statistics Authority of Qatar from 2015 to 2020 [39], a linear correlation has been driven using the curve-fitting tool “Cftool”, available in MATLAB [29] to extrapolate the future population (Figure 1). The projected data generated by Cftool are based on the assumption that population growth will continue to increase at the same rate.

As the food demand is dependent on the interaction between the prices and supply within the food market in Qatar, the demand has increased due to the increase in supply in most of the food categories (Table 2) and the reduced food prices (for example, the producer price change index was −12.9% in 2020 [38]) which has increased the level of willingness of consumers and producers to engage in food buying and selling.

**Figure 2:**
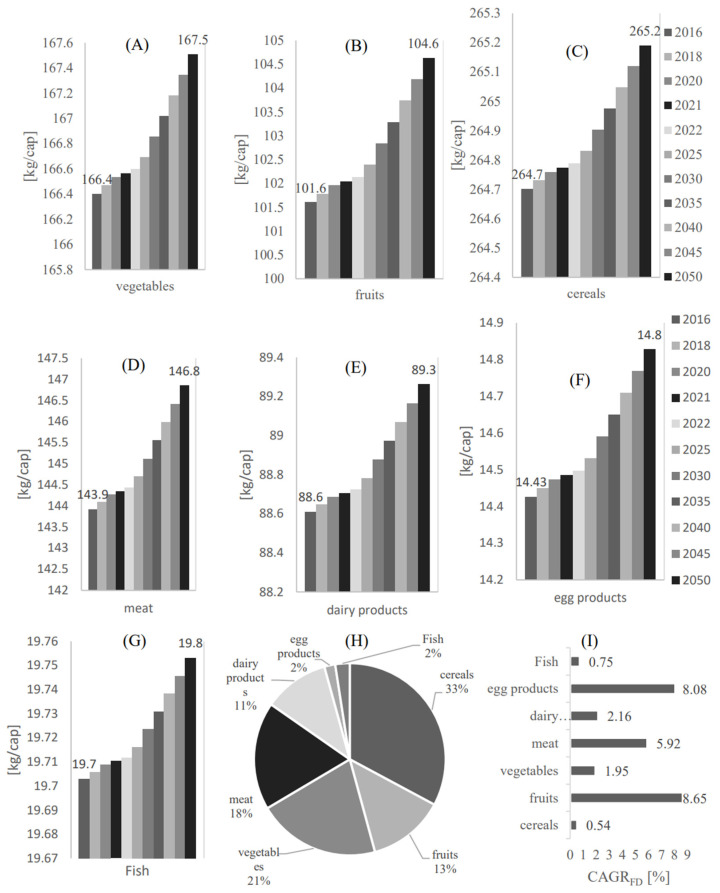
The food demand per capita profile from 2016 to 2050 in Qatar for (**A**) Vegetables, (**B**) Fruits, (**C**) Cereals, (**D**) Meat, (**E**) Dairy products, (**F**) Egg products, and (**G**) Fruit. (**H**) The demand percentage [Mass %] for each food category with respect to the overall food demand. (**I**) The food demand compound annual growth rate.

According to the Planning and Statistics Authority of Qatar [38], the population in Qatar in 2016 was estimated to be 2617634 persons. Therefore, as shown in Figure 2, the data provided in Table 1 for 2016 has been used to estimate the annual food demand per capita for each category, as per Equation 2 [16], where $V_{per\;capita}$, $V_{m}$, and P are the value per capita [kg/cap/year], the measured value [tone/year], and the population [person], respectively.

(2) $$V_{per\;capita}=(V_{m}/P)\times 1000$$

Once the food demand $CAGR_{FD}$ has been estimated, Equation (3) has been used to estimate the consecutive annual food demand ($D_{i+1}$) after 1 year from the year i based on the food demand of the year i ($D_{i}$).

(3) $$D_{i+1}=D_{i}\left(1+CAGR_{FD}\right)$$

### 2.2. Local Food Supply Profiling

Utilizing the raw data of local food production (Table 2) provided by the Planning and Statistics Authority of Qatar from 2016 to 2020 [36], the relative production change factor for each year ($c_{i}$) has been estimated using Equation (4) [17] for all food categories, where $x_{i+1}$ and $x_{i}$ are the local production values for the year i+1 and i, respectively. The average relative production change factor ($\bar{c}$) from 2016–2020 has been estimated using Equation (4) [17].

(4) $$c_{i}=\left[(x_{i+1}/x_{i}\right)-1]\times 100\%$$

(5) $$\bar{c}=\frac{\sum_{i=2017}^{n=2020}c_{i}}{n-i+1}$$

As can be seen in Table 2, the local supply of dairy products has significantly increased due to one of the most important steps taken to achieve a higher level of food security, which resulted in the establishment of Baladna [30], a Qatari agricultural company that raises livestock and produces dairy products. Baladna is an agricultural company that raises livestock and produces dairy products. Over ninety-five percent of Qatar’s fresh dairy products come from this company, making it the biggest locally-owned food and dairy producer in the nation. The sheep and goat farm that would later become what the company was established in [40]. It started manufacturing processed dairy products for the Qatari market in May of 2017 [41] when it was first established.

To estimate the local food supply per capita, the data provided in Table 2 for the local food supply in Qatar in 2016 have been used as per Equation (2) [42].

Once the average relative local production change factor ($\bar{c}$) has been estimated, Equation (6) [43] has been utilized to estimate the projected local food production per capita for each food category through the period of 2020–2050].

(6) $$x_{i+1}=x_{i}\left[\left(\bar{c}\;\%/100\right)\right)+1]$$

Assuming that the needed agricultural area is proportional to the production amount, the average local production change factor ($\bar{c}$) can be used to predict Qatar’s future local agricultural area through Equation (3). The future values of the local production area have been projected by the Planning and Statistics Authority of Qatar from 2016 (Table 3) [36]. The reference [36] data describing the agricultural area designated for green fodder have been assigned to this study’s meat, dairy, and egg products category.

### 2.3. International Food Supply Profiling (Imports) and Food Shares by Origin

As shown in Equation (7) [37,43], the necessary annual international food supply per capita for the year i ($I_{i}$) has been estimated by subtracting the yearly local food supply per capita ($x_{i})$ from the annual food demand ($D_{i}$) that have been assessed as per the methodology of Section 2.1 and Section 2.2 (Equations (3) and (6)), respectively. Once $I_{i}$ has been estimated for the period of [2016–2050], the compound annual growth rate of the international food supply ($CAGR_{FI}$) has been calculated for each food category using Equation (8) [37]. Where $I_{EV}$ and $I_{IV}$ are the end and initial values of the food imports, respectively. The annual and average ratios of the local food supply to the total demand ($\alpha_{i}$) and ($\bar{\alpha }$) are estimated as per Equations (9) and (10). Similarly, the annual and average ratios of the local food supply to the international food supply ($\beta_{i}$) and ($\bar{\beta }$) are estimated as per Equations (11) and (12) [37,43].

(7) $$I_{i}=D_{i}-x_{i}$$

(8) $$CAGR_{FI}={\left(I_{EV}/I_{IV}\right)}^{1/n}-1$$

(9) $$\alpha_{i}=x_{i}/D_{i}$$

(10) $$\bar{\alpha }=\frac{\sum_{i=2016}^{n=2050}\alpha_{i}}{n-i+1}$$

(11) $$\beta_{i}=x_{i}/I_{i}$$

(12) $$\bar{\beta }=\frac{\sum_{i=2016}^{n=2050}\beta_{i}}{n-i+1}$$

### 2.4. Food Self-Sustainability

This section identifies the required action to reach food self-sustainability. To achieve this aim, the entire annual food demand ($D_{i}$) is to be covered by the annual local food production in the future (i.e., from 2025 to 2050). This essentially means that the additional local food supply must replace the entire international food supply (*I_i_*) to achieve food-self sustainability ($x_{i,add})$. To put this into a mathematical context, Equations (7) and (11) can be written as Equations (13) and (14). Dividing Equation (13) by $x_{i,add}$ and substituting $\frac{x_{i}}{x_{i,add}}$ by $\beta_{i}$ yields Equation (15). By introducing the new term ($\gamma_{i}$), which defines the ratio of ($x_{i,add})$ to ($D_{i}$), Equation (15) can be rewritten as Equation (16).

(13) $$x_{i,add}=D_{i}-x_{i}$$

(14) $$\beta_{i}=x_{i}/x_{i,add}$$

(15) $$1=(D_{i}/x_{i,add})-\beta_{i}$$

(16) $$\gamma_{i}=\frac{1}{1+\beta_{i}}$$

### 2.5. Water and Energy for Food

According to [44], the total agricultural water consumption ($V_{tot}$) in Qatar in 2016 was 269.29 MCM. Utilizing the data provided by the reference [45], which reports the virtual water share in Qatar ($V_{w,i}/V_{tot}$) for each food category to link virtual ratios with self-sufficiency levels, the water consumption ($V_{w,i}$) and the average monthly water treatment rates (${\dot{\bar{V}}}_{w,i}$) for each food category can be estimated, as shown in Equation (17) and Table 4. In addition, by integrating the data in Table 2, which describes the local food supply for each category, into Table 4, the water intensity for each food category (${\dot{V}}_{W}$) can be estimated using Equation (18) [46].

(17) $$V_{w,i}=(V_{w,i}/V_{tot})V_{tot}$$

(18) $${\dot{V}}_{W,i}=V_{w,i}/x_{i}$$

According to the reference [47], using Treated Sewage Effluent (TSE) in irrigation is cheaper than desalination. It has been reported that the cost to reuse wastewater is $C_{t}$ = $0.28/m^3^, which is only half of the desalinated water cost [48]. According to the reference [49], the energy for treating sewage effluent $E_{t}$ is 1.2 kWh/m^3^.

Therefore, as a sustainable solution, treated water is assumed to be the source of water for irrigation. It is also assumed social barriers are overcome for the use of treated water and the motivation for this is that:

- Groundwater is in finite supply and depleting;
- Desalinated water is costly and, thus, the cost would be transferred to the consumer when purchasing the food products.

The energy needed for the necessary irrigation of each food category ($E_{i}$), and the energy intensity (energy required per crop mass) ($\dot{E}$) are estimated using Equations (19) and (20) [47,48,49,50], Table 4.

(19) $$E_{i}=E_{t}V_{w,i}$$

(20) $$\dot{E_{i}}=E_{t}{\dot{V}}_{W,i}$$

## 3. Results and Discussion

### 3.1. Food Demand Profiling

Figure 2 shows the food demand per capita profile from 2016 to 2050 in Qatar for (A) Vegetables, (B) Fruits, (C) Cereals, (D) Meat, (E) Dairy products (F), Egg products, and (G) Fruit. As shown in Figure 2A, the food demand in the vegetable food category is expected to reach 167.5 kg/cap in 2050. A similar demand trend to the vegetable category is found in the fruits demand category, which is expected to reach 104.6 kg/cap in 2050. However, among these categories, the demand for cereal will be the highest compared to the other food demand categories, with an expected demand value of 265.2 kg/cap in 2050. In contrast, the demand for egg and fish products is the least compared to the other food demand categories, with an estimated demand value of 14.8 kg/cap and 19.8 kg/cap in 2050, respectively. To provide a governing profile of these food categories, the demand percentage [Mass %] for each food category with respect to the overall food demand and the food demand compound annual growth rates have been estimated in Figure 2H,I, respectively. As shown in Figure 2H, cereals have the highest food demand with a demand percentage of 33% compared to the other food categories. This is followed by vegetables, meat, fruits, dairy products, egg, and fish products with demand percentages of 21%, 18%, 13%, 11%, 2%, and 2%, respectively, compared to the other food categories.

As shown in Figure 2I, fruits have the highest compound annual growth rate compared to the other food categories, with a $CAGR_{FD}$ of 8.65 %. This is followed by eggs, meat, dairy, vegetables, fish, and cereals with a $CAGR_{FD}$ of 8.08%, 5.92%, 2.16%, 1.95%, 0.75%, and 0.54%, respectively. Remarkably, despite having the lowest $CAGR_{FD}$, cereals are still expected to count for the highest food demand among the other food categories in 2050.

### 3.2. Local Food Supply Profiling

**Figure 3:**
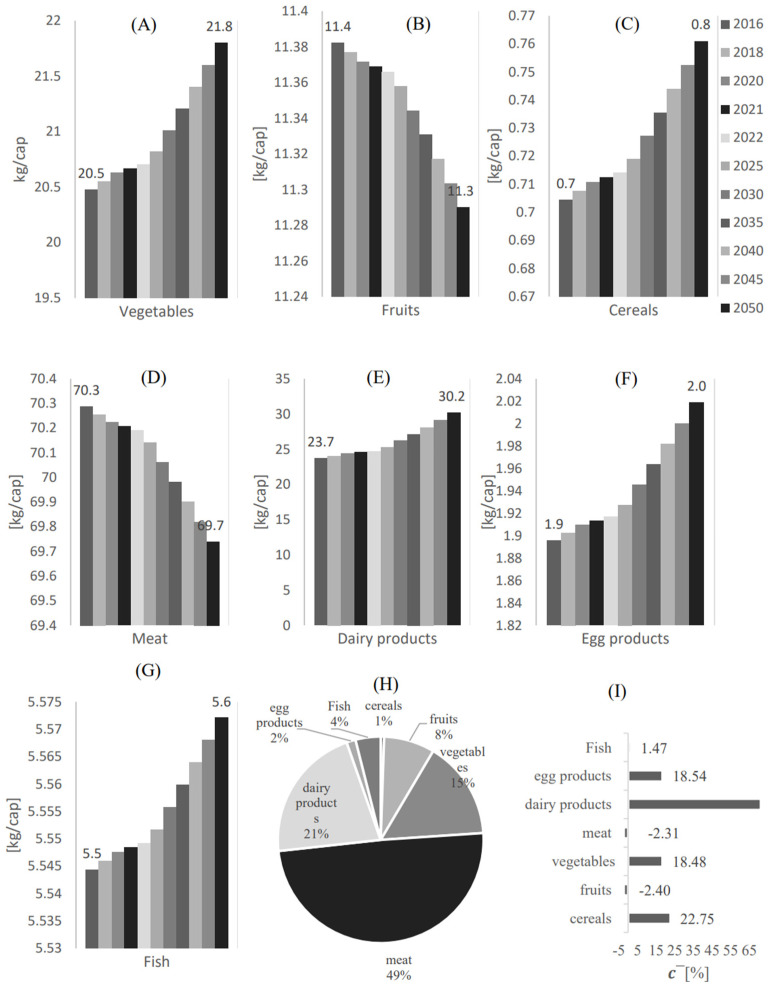
The local food supply per capita profile from 2016 to 2050 in Qatar for (**A**) Vegetables, (**B**) Fruits, (**C**) Cereals, (**D**) Meat, (**E**) Dairy products, (**F**) Egg products, and (**G**) Fruit. (**H**) The local food supply percentage [Mass %] for each food category with respect to the overall local food supply. (**I**) The average local food supply change factor.

Figure 3 shows the local food supply per capita profile from 2016 to 2050 in Qatar for (A) Vegetables, (B) Fruits, (C) Cereals, (D) Meat, (E) Dairy products (F), Egg products, and (G) Fruit.

Despite the expected slight drop from 2016 to 2050, the highest local supply compared to the other food categories is likely for the meat category, with an expected supply of 69.7 kg/cap. This is followed by dairy products, vegetables, fruits, fish, and egg products with the estimated supply of 30.2 kg/cap, 21.8 kg/cap, 11 kg/cap, 5.6 kg/cap, and 2 kg/cap respectively. It can be seen that the local supply of cereals has a positive change factor (increased compared to the previous years). Another significant positive change factor is seen in dairy products, scoring approximately 71.3%, Figure 3I. This is followed by cereals, egg products, vegetables, and fish, with change factors of 22.75%, 18.54%, 18.48%, and 1.27%, respectively.

**Figure 4:**
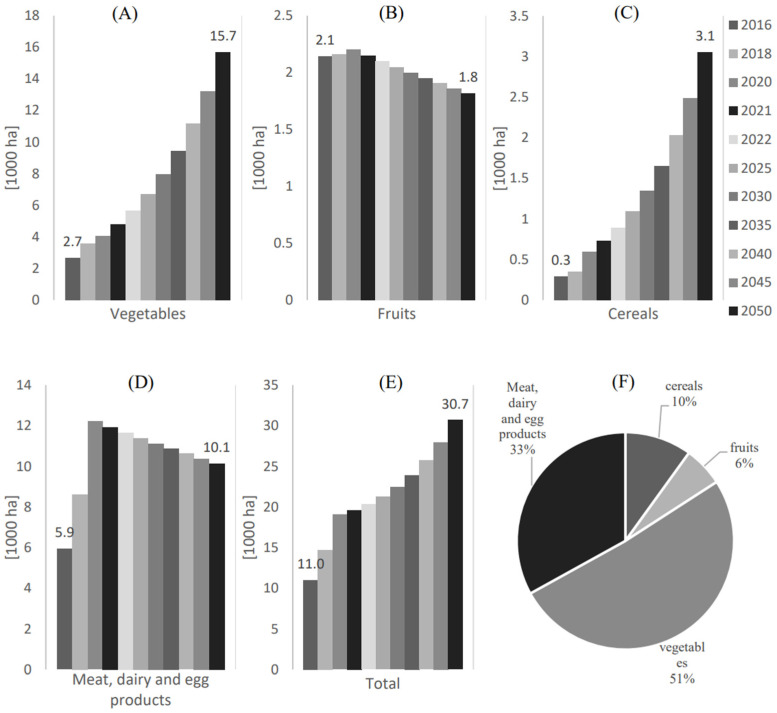
Local agricultural area in Qatar from 2016 to 2050 in Qatar for (**A**) Vegetables, (**B**) Fruits, (**C**) Cereals, (**D**) Meat, dairy, and egg products, and (**E**) Total area. (**F**) The local agricultural percentage for each food category with respect to the total in 2050.

The corresponding areas used for local food supply are shown in Figure 4. As shown in Figure 4A, the largest area is reserved for producing vegetables, with an estimated area increase from 2.7 [1000 ha] to 15.7 [1000 ha] from 2016 to 2050, respectively. This is followed by the area designated for producing meat, dairy, and egg products, estimated to be 10.1 [1000 ha] in 2050.

### 3.3. International Food Supply Profiling

Amongst the positive indicators, dairy products and vegetables have shown a negative import growth factor. This essentially means that the reliance on the imports of these food categories is expected to be reduced; however, in relatively low ratios (Figure 3H).

**Figure 5:**
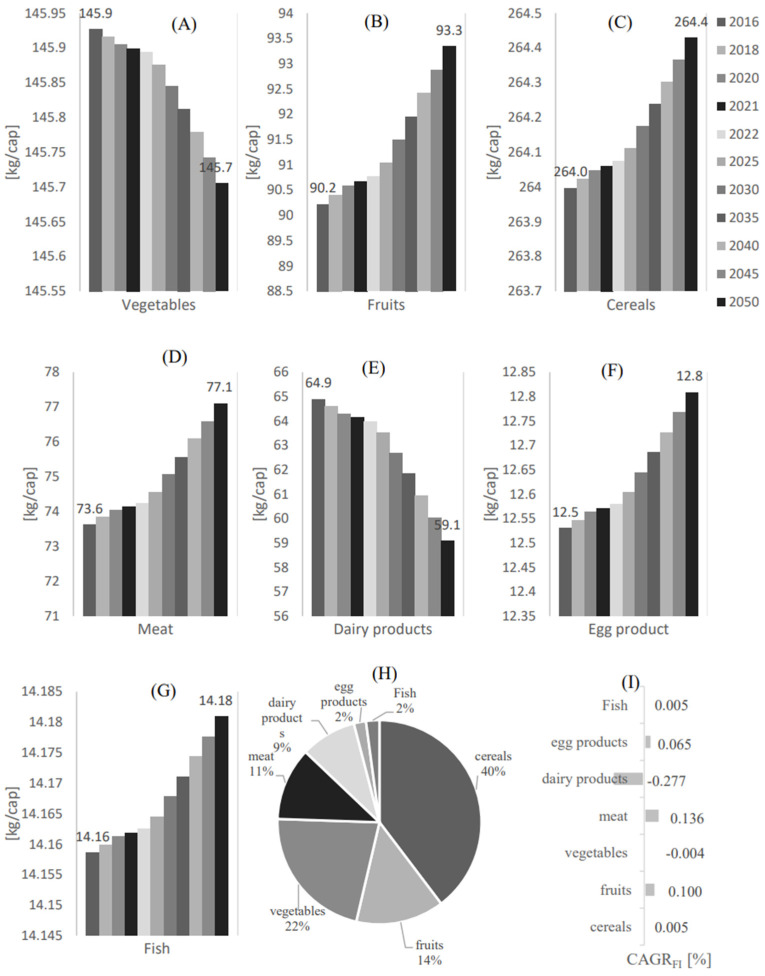
International food supply per capita profile from 2016 to 2050 in Qatar for (**A**) Vegetables, (**B**) Fruits, (**C**) Cereals, (**D**) Meat, (**E**) Dairy products, (**F**) Egg products, and (**G**) Fruit. (**H**) The international supply percentage [Mass %] for each food category with respect to the overall international supply. (**I**) The international food supply compound annual growth rate.

**Figure 6:**
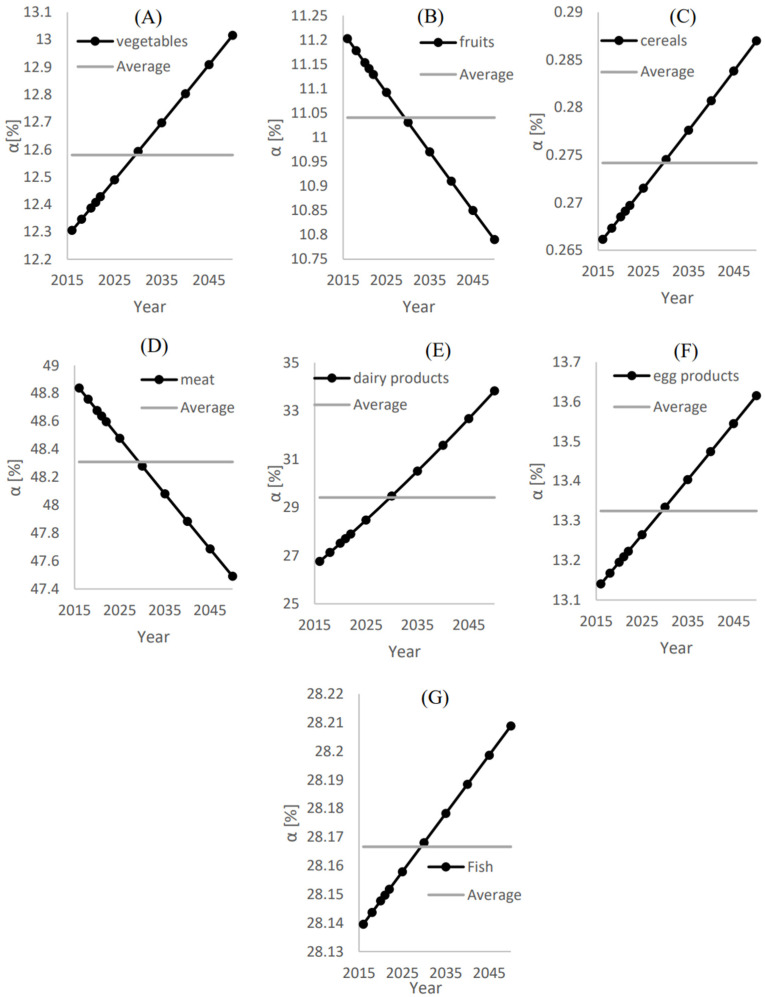
The annual and average ratios of the local food supply to the total demand ($\alpha_{i}$ ) and ($\bar{\alpha }$) for (**A**) Vegetables, (**B**) Fruits, (**C**) Cereals, (**D**) Meat, (**E**) Dairy products, (**F**) Egg products and (**G**) Fruit.

**Figure 7:**
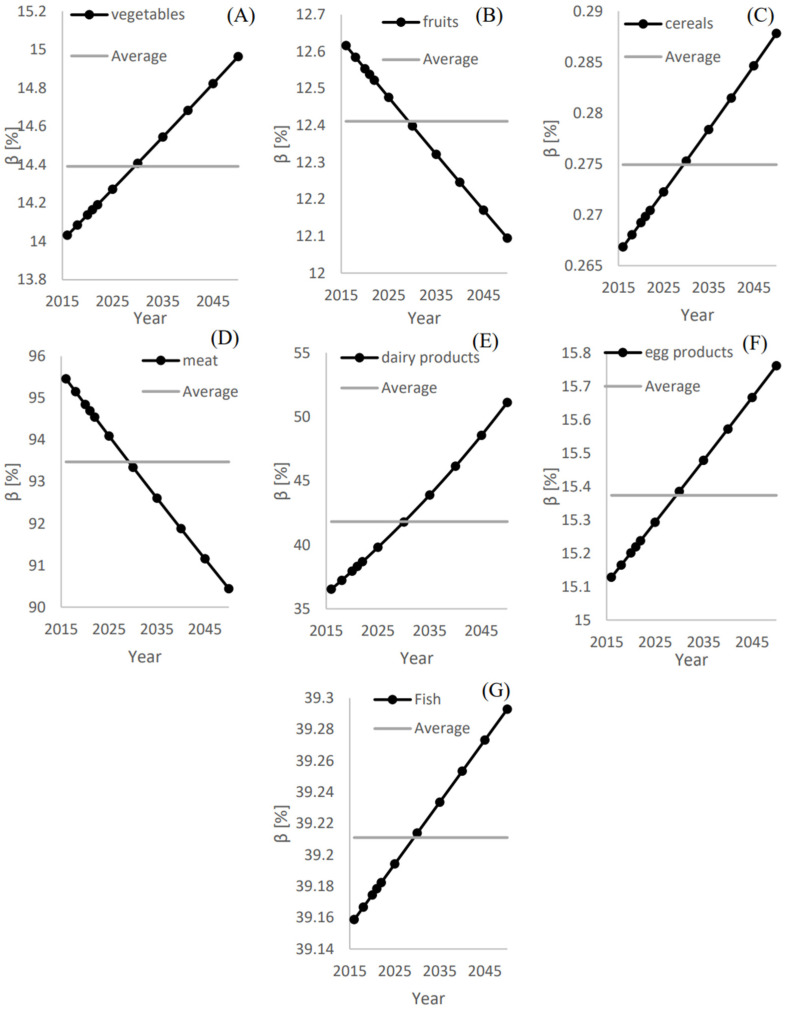
The annual and average ratios of the local food supply to the international supply ($\beta_{i}$ ) and ($\bar{\beta }$) for (**A**) Vegetables, (**B**) Fruits, (**C**) Cereals, (**D**) Meat, (**E**) Dairy products, (**F**) Egg products and (**G**) Fish.

**Figure 8:**
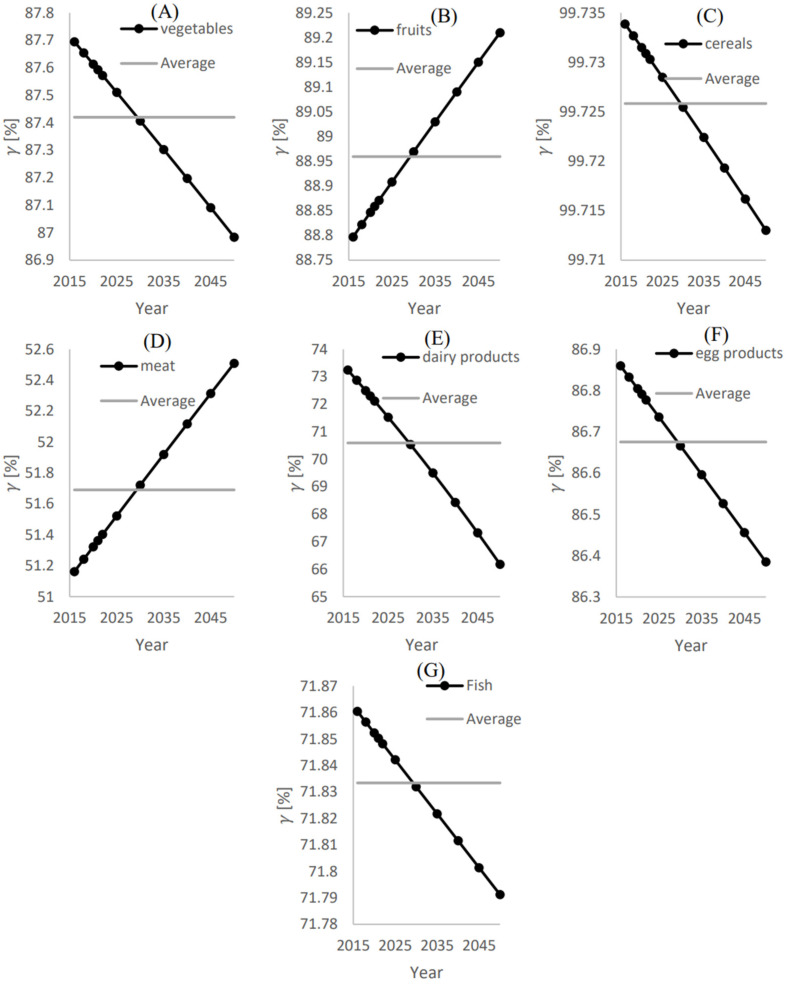
The annual and average ratios of the additional local food supply to achieve food-self sustainability ($x_{i,add})$ to the local demand (γ) for (**A**) Vegetables, (**B**) Fruits, (**C**) Cereals, (**D**) Meat, (**E**) Dairy products, (**F**) Egg products, and (**G**) Fish.

A comparison between the local and international food supply is further highlighted in Figure 5, Figure 6, Figure 7 and Figure 8.

Figure 6 shows the annual and average ratios of the local food supply to the total demand ($\alpha_{i}$) and ($\bar{\alpha }$) for (A) Vegetables, (B) Fruits, (C) Cereals, (D) Meat, (E) Dairy products (F), Egg products, and (G) Fruit. The highest average ratio of the local food supply to the total demand ($\alpha_{i}$) was found for the meat category, which is estimated to be 48.3%. This means that the local production of meat can fulfill 48.3%, as shown in Figure 6D. Although the annual ratio of the local food supply to the total demand ($\alpha_{i}$) is reduced with time, the reduction rate is insignificant.

The second highest local food supply to the total demand ratio is secured by dairy products, as the local supply covers approximately $\bar{\alpha }=$ 29.5% of the total demand for this food category. This is followed by fish, egg products, vegetables, and fruits, with the average local food supply to the total demand ratios of 28.175%, 13.35%, 12.6%, and 11%, respectively.

As shown in Figure 7D, the local meat supply approximately matches the imports with $\bar{\beta }$ = 93.5%. This is followed by dairy products, fish, egg products, vegetables, and fruits with $\bar{\beta }$ of 42%, 39.2%, 15.3.5%, 14.4%, and 12.4%, respectively.

Figure 8 shows the annual and average ratios of the additional local food supply to achieve food self-sustainability ($x_{i,add})$ to the local demand (γ) for (A) Vegetables, (B) Fruits, (C) Cereals, (D) Meat, (E) Dairy products, (F) Egg products, and (G) Fish. As shown in Figure 8D, to achieve food self-sustainability, the local meat supply shall be increased by approximately 51.7% of the required demand. As γ for the meat category is the least compared to the other food categories, this category requires the least effort to achieve food-self sustainability

Nevertheless, the feasibility of increasing the local supply to match the demand is related to the food demand level of each category. In addition, it depends on the water and energy demands, which vary from one food category to another. These parameters are evaluated in Section 3.4 to assess the feasibility of achieving the food self-sustainability criteria of each food category.

### 3.4. Water and Energy Profiling

**Figure 9:**
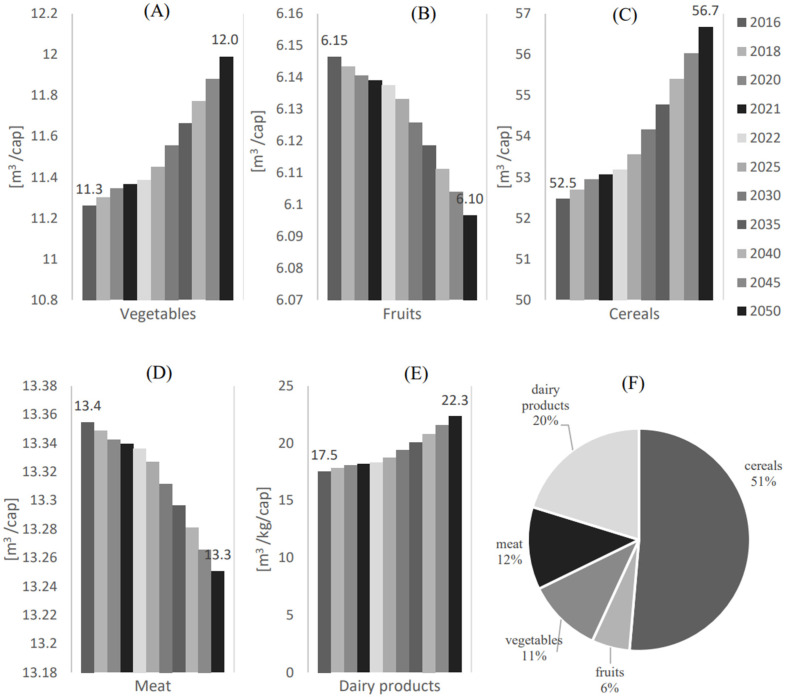
Water consumption ($V_{w,i}$) per capita [$m^{3}/\mathrm{cap}$] for the local food supply of (**A**) Vegetables, (**B**) Fruits, (**C**) Cereals, (**D**) Meat, and (**E**) Dairy products. (**F**) Water consumption share ($V_{w,i}/V_{tot}$) for each food category.

Figure 9 shows the water consumption ($V_{w,i}$) per capita [$m^{3}/\mathrm{cap}$] for the local food supply of (A) Vegetables, (B) Fruits, (C) Cereals, (D) Meat, (E) Dairy products. (F) shows the water consumption share ($V_{w,i}/V_{tot}$) for each food category. The corresponding water shares for dairy products and meat are 20% and 12%, respectively. However, the dairy products and meat categories fulfill significant demand portions, with average ratios of the local food supply to the total demand ($\bar{\alpha }$) of 29.4% and 48.3%, respectively. The local supplies of vegetables and fruits categories consume the least amounts of water, with a water consumption ($V_{w,i}$) of 12 m^3^/cap and 6.10 m^3^/cap in 2050, respectively. The corresponding water shares for the local supplies of vegetables and fruits are 11% and 6%, respectively.

As shown in Figure 5B and Figure 6A, with these water consumption rates of the local supplies of vegetables and fruits, 12.6% and 11.05% of the demand for these food categories can be fulfilled, respectively.

**Figure 10:**
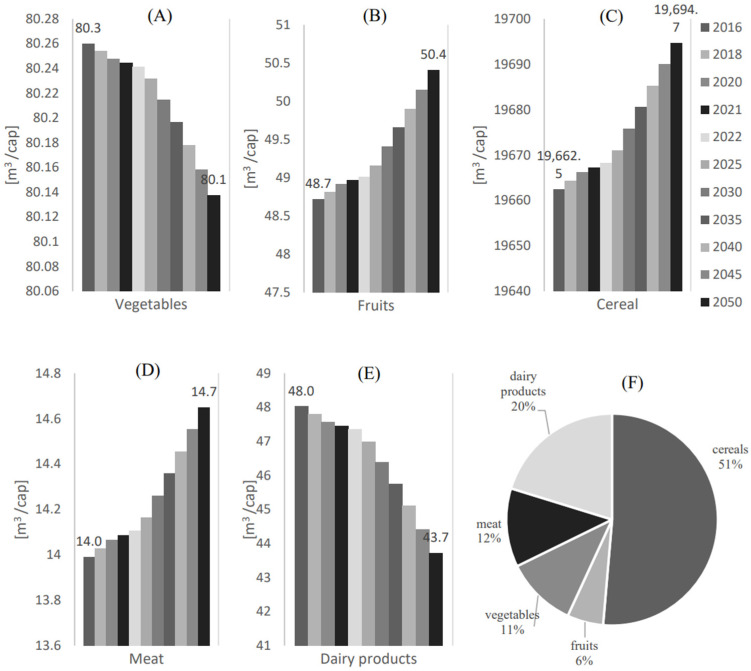
Water consumption ($V_{w,i}$) per capita [$m^{3}/\mathrm{cap}$] for the additional local food supply to achieve food self-sustainability ($x_{i,add})$ for (**A**) Vegetables, (**B**) Fruits, (**C**) Cereals, (**D**) Meat, (**E**) Dairy products. (**F**) Water consumption share ($V_{w,i}/V_{tot}$) for each food category.

Figure 10 shows water consumption ($V_{w,i}$) per capita [$m^{3}/\mathrm{cap}$] for the additional local food supply to achieve food self-sustainability ($x_{i,add})$ for (A) Vegetables, (B) Fruits, (C) Cereals, (D) Meat, and (E) Dairy products. (F) shows the water consumption share ($V_{w,i}/V_{tot}$) for each food category. For the meat category, Figure 10D suggests that fulfilling the water demand to achieve the food self-sustainability of this category is expected an estimated water consumption of 14.7 m^3^/cap in 2050. Another potential for achieving food self-sustainability is found for dairy products and fruits, with an estimated water consumption for the additional local food supplies of 43.7 m^3^/cap and 50.4 m^3^/cap in 2050, respectively.

Although the estimated water consumption ($V_{w,i}$) for the additional local supply of vegetables to achieve food self-sustainability ($x_{i,add})$ is relatively high (80.3 m^3^/cap −80.1 m^3^/cap in 2016–2050) compared to the meat, fruits, and dairy products, it could be still expected to be within the food self-sustainable categories due to its low water consumption rates (i.e., the second lowest water consumption share compared to the other categories ($V_{w,i}/V_{tot}$ = 11%)).

**Figure 11:**
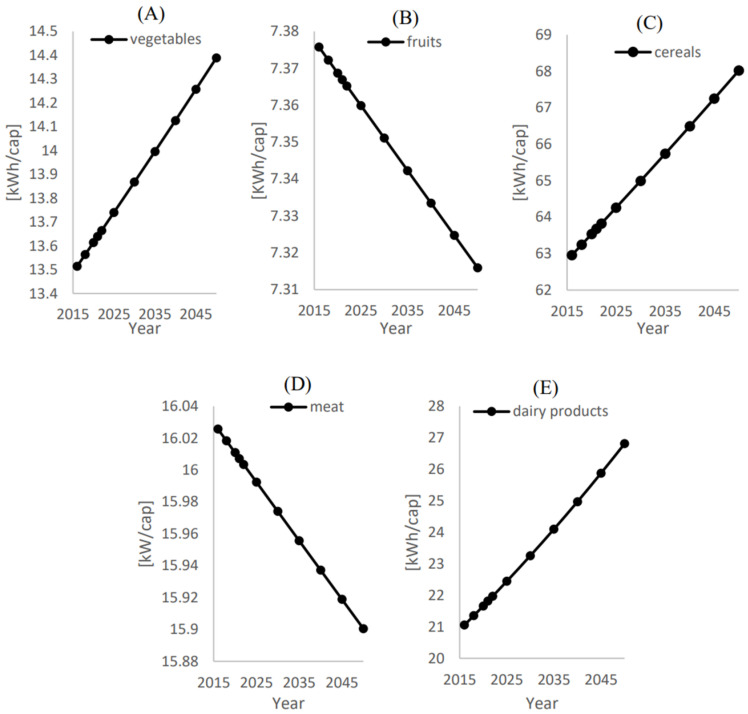
Energy consumption ($E_{i}$) per capita [kWh/cap] for local food supply ($x_{i})$ for (**A**) Vegetables, (**B**) Fruits, (**C**) Cereals, (**D**) Meat, and (**E**) Dairy products.

**Figure 12:**
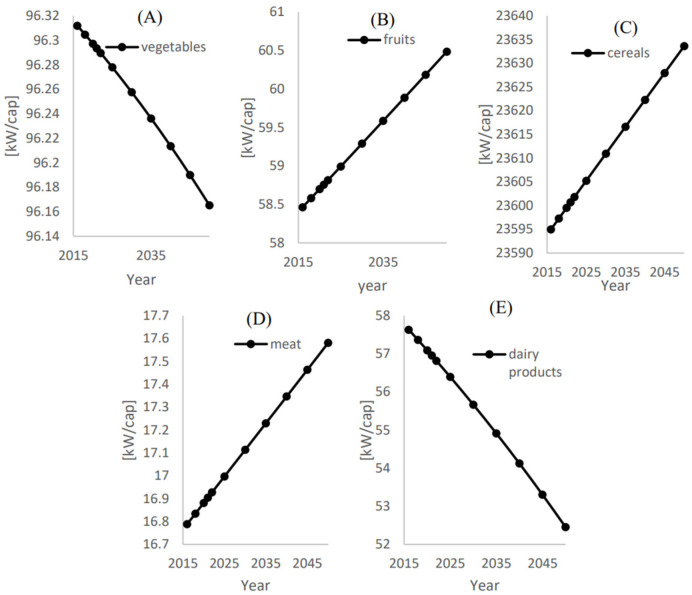
Energy consumption ($E_{i}$) per capita [kWh/cap] for the additional local food supply to achieve food self-sustainability ($x_{i,add})$ for (**A**) Vegetables, (**B**) Fruits, (**C**) Cereals, (**D**) Meat, (**E**) Dairy products.

As the energy quantifications correspond to the water consumption shown in Figure 9 and Figure 10, energy trends follow the same water consumption patterns. However, the energy quantification can be obtained in Figure 11 and Figure 12. Figure 11 shows that the corresponding energies of the treated water to be used in irrigation for the local supply of vegetables, fruits, cereals, meat, and dairy products are 14.38 kW/cap, 7.31 kW/cap, 68 kW/cap, 16.1 kW/cap, and 26.81 kW/cap in 2050, respectively.

Figure 12 shows that the corresponding energies of the treated water to be used in irrigation for the additional supply to achieve food self-sustainability ($x_{i,add})$ of vegetables, fruits, cereals, meat, and dairy products are 96.16 kW/cap, 60.5 kW/cap, 23.633 MW/cap, 17.58 kW/cap, and 52.45 kW/cap, respectively.

## 4. Discussion and Conclusions

Following the below strategic, customized recommendation for each food category to reach food sustainability in Qatar will increase local and external investors’ willingness to achieve a higher food sustainability and security level. Secure access to food can produce wide-ranging positive impacts, including economic growth, job creation, and trade opportunities.

- For cereals, to secure strategic cereal reservoirs to increase the food security level of this category. The capacity of these reservoirs could be sized to match the additional required local food ($x_{i,add})$ within a period that is defined by the production-to-expiry dates of the cereal products).
- For the meat, vegetables, fruits, and dairy products, it is recommended to size water treatment plants to fulfill the water demands for the existing local supplies ($x_{i}$ ) as well as for the to-be added food supplies to achieve food self-sustainability ($x_{i,add})$.

The basis of these recommendations is detailed through this paper which evaluates the Energy–Water–Food Nexus Profile of Qatar on a superstructural level. The storyline of BAU assumes a continuation of the historical development trend, including additional measures with limited effects on energy efficiency improvement, renewable energy deployment, and electrification of end-uses [35]. Therefore, to apply the BAU storyline, trends of past data have been used to provide future projections for 2050 using the statistical prediction tools such as the compound annual growth rates of food demand ($CAGR_{FD}$), international supply ($CAGR_{FI}$), and the average local food supply change factor ($\bar{c}$). Once the BAU storyline has been generated, the source-to-demand correlations have been defined for each food category. Such correlations include the annual and average ratios of the local food supply to the total demand (i.e., $\alpha_{i}$ and $\bar{\alpha }$) and the ratios of the local food supply to the international supply (i.e., $\beta_{i}$ and $\bar{\beta }$). In addition, as an effort to identify the required action to reach food self-sustainability, the additional local food supply to achieve ($x_{i,add})$ and its ratio to the local demand (γ) have been defined. Finally, utilizing the virtual water share in Qatar ($V_{w,i}/V_{tot}$) and the total agricultural water consumption ($V_{tot}$) reported for each food category, the water consumption ($V_{w,i}$) for each food category has been estimated. As a sustainable solution recommended in the literature [31,32,33], using TSE in irrigation has been adopted in this study; thus, the energy required to reuse wastewater in irrigation to achieve food self-sustainability has been estimated in this paper.

The highest average ratio of the local food supply to the total demand ($\alpha_{i}$) was found for the meat category, which was estimated to be 48.3%. This essentially means that the local production of meat is capable to fulfill 48.3%. The local meat supply approximately matches the imports with $\bar{\beta }$ = 93.5%. This is followed by dairy products, fish, egg products, vegetables, and fruits with $\bar{\beta }$ of 42%, 39.2%, 15.3.5%, 14.4%, and 12.4%, respectively. To achieve food-self sustainability, the local meat supply shall be increased by approximately 51.7% of the required demand. As γ for the meat category is the least compared to the other food categories, this category requires the least effort to achieve food self-sustainability.

For the meat category, the results show that fulfilling the water demand to achieve this category’s food self-sustainability is expected with an estimated water consumption of 14.7 m^3^/cap in 2050. Another potential for achieving food self-sustainability is found for dairy products and fruits, with an estimated water consumption for the additional local food supplies of 43.7 m^3^/cap and 50.4 m^3^/cap in 2050, respectively. Although the estimated water consumption ($V_{w,i}$) for the additional local supply of vegetables to achieve food self-sustainability ($x_{i,add})$ is relatively high (80.3 m^3^/cap −80.1 m^3^/cap in 2016–2050) compared to the meat, fruits, and dairy products, it could be still expected to be within the food self-sustainable categories due to its low water consumption (i.e., the second lowest water consumption share compared to the other categories ($V_{w,i}/V_{tot}$ = 11%).

Therefore, due to the high feasibility (relatively low water and energy consumption) of producing vegetables, fruits, meat, and dairy products, it is recommended to size water treatment plants to fulfill the water demands for the existing local supplies ($x_{i}$) as well as for the to-be added food supplies to achieve food self-sustainability ($x_{i,add})$, which are both defined in this paper.

## Figures and Tables

**Table 1:** Food demand in Qatar from 2015 to 2020 [37].

Food Category	Year						$\mathrm{Food}\;\mathrm{Demand}\;{CAGR}_{FD}\;[\%]$
	2015	2016	2017	2018	2019	2020	
Cereals [tone]	478,022	692,891	410,644	586,720	561,578	491,140	0.543
Fruits [tone]	187,912	265,962	221,651	274,004	288,833	284,471	8.647
Vegetables [tone]	425,649	435,583	338,803	512,863	474,180	468,688	1.945
Meat [tone]	173,665	376,732	232,597	233,803	304,136	231,576	5.924
Dairy products [tone]	248,392	231,946	209,458	334,412	274,481	276,353	2.156
Egg products [tone]	32,526	37,762	42,412	44,387	50,383	47,967	8.079
Fish [tone]	47,069	51,575	48,426	49,243	53,382	48,855	0.748

**Table 2:** Local food supply in Qatar from 2016 to 2022 [36].

Food Category	Year					$\bar{c}\;[\%]$
	2016	2017	2018	2019	2020	
Cereals [tone]	1844	2573	3106	1980	3307	22.75413
Fruits [tone]	29,795	28,975	29,277	26,400	26,914	−2.39743
Vegetables [tone]	53,599	55,579	74,650	91,470	103,693	18.47552
Meat [tone]	183,988	24,805	36,036	32,555	46,124	−2.30513
Dairy products [tone]	62,061	56,146	226,408	199,926	206,683	71.35022
Egg products [tone]	4962	5753	8372	7943	9358	18.53886
Fish [tone]	14,513	15,358	14,665	16,938	15,087	1.470364

**Table 3:** Local agricultural area in Qatar in 2016 [36].

Food Category	Designated Local Agricultural Area [1000 ha]
Cereals	0.3
Fruits	2.1
Vegetables	2.7
Meat, dairy, and egg products	5.9
Total	11.0

**Table 4:** The water treatment rates and water consumption shares for each food category in 2016.

Food Category	$V_{w,i}/V_{tot}$ [%] [44]	$V_{w,i}$ $\left[\mathrm{Million}\;m^{3}\right]$ [44]	${\dot{\bar{V}}}_{w,i}$ $[\mathrm{Million}\;m^{3}/\mathrm{Month}]$ [44]	$x_{i}$ [1000 kg] [36]	${\dot{V}}_{W,i}$ [m^3^ Water/kg Crop]	$\dot{E_{i}}$ [kWh/kg Crop]
Cereals and flour	51	137.34	11.45	1844	74.48	89.376
Fruits and sugar	6	16.16	1.35	29,795	0.54	0.648
Vegetables	11	29.62	2.47	53,599	0.55	0.66
Meat	13	35.01	2.9	183,988	0.19	0.228
Dairy products	17	45.78	3.81	62,061	0.74	0.888
Egg products	0	0.00	0.00	4962	0.00	0
Fish	0	0.00	0.00	14,513	0.00	0

Note: The total agricultural water consumption in Qatar in 2016 was 269.29 MCM [18].

## Data Availability

Data is contained within the article.
